# Notes and News

**Published:** 1889-01-26

**Authors:** 


### Notes and News.

Collected and Communicated.

The annual report of the Liverpool Dental Hospital for 1888 is issued, and tells of no less than 25,000 unfortunates who were last year driven by pain to endure the heroic remedy of "having it out." We condole with the victims and congratulate the hospital.

A concert, by nurses and friends, was given to the patients of the Royal South Hants Infirmary as a New Year's treat. Some excellent glees were sung by the nurses, conducted by the Rev. S. W. Stevens; and Dr. and Miss Aldridge also lent their aid. Nurse Potter gained an encore for one of her songs; and the amusing operetta, "The Blind Beggars," was most heartily applauded.

Miss Gladstone writes to the *Times* to thank those who have responded to her mother's appeal for funds for the Woodford Convalescent Home. Patients are received at this Home free for a two weeks' stay, if recommended by a clergyman or member of the Charity Organisation Society. It is not generally known that blind convalescents are also taken at Woodford. Mrs. Gladstone does much for the Home, but is obliged sometimes to appeal to the public for aid.

Another complaint against a hospital, and this time it is the Wigan Infirmary which is attacked. Mr. Hurst declares that on January 5th a man named Shearden was run over by a goods train and taken to the Wigan Infirmary, which he reached at 3.30 p.m. There was no doctor in attendance, and the nurses could not agree whose visiting day it was, so an hour or more elapsed ere the unfortunate patient received attention. The victim died next day. We wait the explanation of this last "charge against a hospital."

Her Royal Highness Princess Christian, accompanied by Dr. Cohn, of Wiesbaden, and attended by Mrs. Jeune, honoured the Queen Charlotte's Lying-in Hospital with an unexpected visit on Monday afternoon last. Her Royal Highness visited a number of the wards, and also inspected the kitchen arrangements and the nurses' and servants' halls. At the termination of the visit, which lasted about an hour, her Royal Highness was pleased to express her satisfaction with everything she had seen.

The Children's Orchestra, of which H.R.H. the Duchess of Teck is president, will give its first concert of the present season on Wednesday evening, January 30th, at the Westminster Town Hall. The proceeds will be given to the Newport Market Industrial School for Boys, Coburg Row, Westminster. Lady William Lennox and other ladies are interesting themselves in the sale of tickets, which range between 7s. 6d. and 2s. 6d. Captain the Hon. Randolph Stewart and Mr. Percy Armitage are also taking an active part. It is much to be hoped that the ladies and gentlemen who are devoting their time and energy to the laudable work of helping the Newport Market School will be enthusiastically supported.

A delightful Doll Show has been held at Cork in aid of the Hospital for Women and Children. There was a hospital stall, where ward scenes were represented and nurse-dolls were on sale; there was a special stall for dolls dressed by children, and also a beauty show stall. The Countess of Bandon distributed the prizes. Owing to the depressed state of affairs in Ireland it is impossible to maintain the hospital without appealing to outsiders, and therefore a subscription list has been opened to clear off the building debt of £300. The doll show cleared £80, which goes towards a deficit of £150 in the current expenditure. Help earnestly requested.

The *Oldham Evening Express* of January 10th contained an interesting article on the infirmary of that town. The matron, Miss Thompson, took the interviewer round the wards and over the whole building, and wherever he went that interviewer was struck by the largeness and cleanliness of everything. On leaving the infirmary, amongst the list of contributions the interviewer notices that of the Oldham colliers, which is considerable—£122 11s. 4d. This says all that is necessary in praise of the infirmary, for the poor colliers, who often meet with serious and awful accidents, must be best able to judge of the efficiency of the institution where they seek help when suffering.

At the last meeting of the Council of the National Pension Fund, Mr. Walter H. Burns in the chair, the manager reported that the number of applications for pensions and sick pay up to date was 637 (exclusive of the Mildmay nurses, numbering about 100), of whom 501 had paid their contributions, amounting to £8,930, of which £2,784 has been paid in since the last monthly meeting; 63 applications had been received during the corresponding period; and 57 were accepted at the present meeting (exclusive of the Mildmay nurses). It was reported that the funds invested amounted to upwards of £33,700.

It seems that the duties of presidents to charities chiefly include subscribing. Speaking of the financial condition of the Nottingham Hospital for Epilepsy and Paralysis, the *Nottingham Evening News* points out the number of wealthy presidents of that institution, and concludes:—"It is by no means creditable to the peers who have signed this appeal to come begging before the public when they are themselves quite capable of providing all the funds the institution needs. Of what use, we may ask, is such a formidable array of vice-presidents, if their generosity does not extend beyond giving a few paltry pounds a-year and lending their names and patronage?" Perhaps the editor of the *Evening News* has not considered that it is the public, not the presidents, who benefit by the hospital.

A Correspondent writes: The annual entertainment to the nursing staff of St. George's Hospital on January 10th and 11th was this year more than ordinarily successful. Two short dramatic pieces—*In Honour Bound* and *Anything for a Change*—were very cleverly acted by Mr. and Mrs. Frederick Upton and friends, whose kindness in giving performances of this kind in hospitals and other institutions is well known and highly appreciated by their numerous audiences. Between the two plays an interval was very pleasantly filled by some nurses, who sang glees (for female voices), trios, duets, and solos in a manner which was highly creditable, especially when we consider how short a time nurses can spare from their hours of recreation for musical practices. The entertainment was given in the board-room, and the large audience included several members of the staff and committee, as well as friends of the nurses, each nurse being allowed to invite one friend. Refreshments were served afterwards in the committee-room, and at ten o'clock a very enjoyable evening was brought to a close.

THE following vacancies are announced this week, the amount of salary in each case being given after the name of the institution :—

**Resident Medical Officers.**

State Criminal Lunatic Asylum, Broadmoor. Junior Assistant Medical Officer.—£175, board, etc.
Royal Albert Edward Infirmary, Wigan. Junior House Surgeon. —£80, board, etc.
York Retreat for Insane Members of the Society of Friends. Junior Assistant Medical Officer.—£80, board.
Bury Union, Lancaster. District Medical Officer.—£70.
Evelina Hospital for Sick Children. House Surgeon.—£70, board, etc.
Lunatic Hospital, The Coppice, Nottingham. Assistant Medical Officer.—£100, board, etc.
Somerset and Bath Asylum, Wells. Junior Assistant Medical Officer.—£100, board, etc.
Birmingham Workhouse Infirmary. Clinical Assistant.—Board, etc.
Birmingham General Hospital. Assistant House Surgeon.—Board, etc.
Guy's Hospital. Six Assistant Dental Surgeons.
Jaffray Hospital, Birmingham. Resident Medical Officer.—£150, board, etc.

**Non-resident Medical Officers.**

Great Northern Central Hospital. Physician to Out-patients.
Farringdon General Dispensary, Holborn. Honorary Physician.

A NEW YEAR'S entertainment and Christmas tree were given at the National Orthopædic Hospital (for the Deformed), Great Portland Street, on Saturday week, by friends to the hospital. All the patients, including men, women, and children, had tea in the board-room, the tables being prettily decorated with plants and fruit, and the room festooned with flags and Chinese lanterns. After the tea readings were given by Mrs. Becker, and music by Mrs. Becker and other ladies. Songs, concertina and pianoforte performances followed. A very large Christmas tree, covered with several pretty and useful presents for each patient, was a chief feature of the occasion. The evening passed off most successfully, and all were pleased.

THE annual tea and entertainment of the West End Hospital for Nervous Diseases, the expenses of which were defrayed by a special fund raised for the purpose by the matron, took place on Wednesday, January 9th, at Welbeck Hall, W. The matron, Mrs. Honeyman Brown, assisted by Miss Thompson, worked energetically to make it a success, and their efforts were well rewarded. There were upwards of two hundred children and patients present. After tea the entertainment commenced with the distribution of toys, etc., from the Christmas tree, and in addition to songs, recitatione, etc. (contributed by many friends), there was a fancy quadrille dance in Kate Greenaway costume, Mrs. Jarley's Waxwork, Haydn's Joy Symphony, ventriloquism, and conjuring. The medical staff were represented by Dr. Tibbits, Dr. Winslow, Dr. Herschell, and Mr. Benton.

ON Wednesday evening, January 2nd, the annual Christmas treat to the patients and servants of the Central London Throat and Ear Hospital, Gray's Inn Road, took place. The entertainment consisted of the usual Christmas games, songs, and dancing, and all present thoroughly enjoyed themselves. The musical part of the entertainment was under the direction of Mr. J. S. Pearson and his daughter, assisted by several friends, and last, but not least, the "Excelsior Quartet." On the following evening a special treat was given to all the children who had been in the hospital during the past year, and there were at least fifty present. For them there was a Christmas tree provided, and Mr. Middleton amused them with a magic lantern. The Rev. S. Vercoe, senior curate of St. Jude's, assisted the matron with the distribution of the toys. For the last three years the success of these entertainments has been entirely due to the untiring devotion and energy of Miss Danter, the matron to the hospital, whose sympathy with her work and interest in the welfare of the patients has earned for her the esteem of all who come into contact with her.

AT the meeting of the Metropolitan Asylums Board on January 12th, Mr. Jebb reported that the returns of the small-pox hospitals for the three months ending with the last year showed that there had been but one case admitted, which was from Paddington, and that the wards had otherwise been empty. In regard to fever in the last three months of the year, there were 967 cases at the beginning of the quarter (October) under treatment. During the three months 1,137 were admitted, 209 died, 1,042 were discharged, and 853 remained under treatment. The prevalence of measles came to the fore, and Mrs. Lawrie, a nominated member, moved that the managers should take the initiative, and should open some of the hospitals for the reception of such cases. Dr. Felce (Paddington) seconded the motion. The subject was finally referred to the General Purposes Committee. Mr. Robinson condemned the action of the Ambulance Committee, who, trying to close Hampstead Hospital, sent patients a drive of seven miles to Deptford.

ONE of the most successful entertainments of the hospital Christmas season was that given at St. Mary's on the 10th inst. In the first place there was Mr. Charles Collette, who can rattle off a string of absurdities with such a grave and convincing air of veracity that he forces smiles—no, loud laughter—from the saddest and most serious. He never seems to want time to breathe or invent, but he had frequent pauses forced on him at St. Mary's by the applause which so often stopped the progress of his narrative. After him came a series of admirable magic lantern pictures, depicting some of the most interesting and beautiful of English scenery, with a descriptive narrative by Mr. E. Ash. Finally, there was a short but admirable concert. It is invidious to praise one where all did well, and there was no item which was not fully appreciated by the audience of patients and nurses; but we cannot refrain from saying that the rendering by Miss Nettie Wood, R.A.M., of "La Bouquetière," and the ever delightful "Should He Upbraid," were worth going into hospital to hear. The evident pleasure that was felt by all present must have been a full reward for all their labours in getting up the entertainment—to Miss Medill, the matron of St. Mary's; Dr. Mitchell Bird, the medical superintendent; and Mr. Thomas Ryan, the energetic secretary.

THE opening of the Birmingham Workhouse Infirmary was an affair of magnitude, but Mr. Chamberlain's speech was the chief attraction of the day. It is not often that such a company of celebrities sit down to lunch in a Union Infirmary, and perhaps it was only natural that the speeches should have wandered from the theme of sickness, and turned on the merits of the House of Lords. Lord Norton and Mr. Rathbone, M.P,, also spoke, the first in a most amusing manner, and, altogether, the gathering was a great social success. The principal block of the new building had been handsomely decorated, and a remarkably pretty effect had been secured in several apartments of it, despite the fact that its walls are absolutely bare and featureless. A portion of the corridor, which served as an entrance hall, was draped with tapestries, and set off with flower-beds and tall ferns. One of the wards was used as the luncheon-room. The brick walls and the pillars had been covered with curtains of Indian muslin, chenille, and lace, and there was an abundance of bold and suitable mottoes, flags, and shields; while the tables were profusely decked with flowers, and the picturesque simplicity of the costumes of the nurses, who acted as waitresses, imparted a pleasing novelty to the scene. An interesting circumstance in connexion with the mottoes and shields is that they were the work of an aged inmate of the workhouse, who is still an enthusiastic designer and draughtsman.

